# An evaluation of obstetric ultrasound education program in Nepal using the RE-AIM framework

**DOI:** 10.1186/s12909-021-02484-3

**Published:** 2021-01-15

**Authors:** Jieun Kim, Prabin Raj Shakya, Sugy Choi, Joong Shin Park, Suman Raj Tamrakar, Jongho Heo, Woong-Han Kim

**Affiliations:** 1 JW LEE Center for Global Medicine, Seoul National University College of Medicine, Seoul, Republic of Korea; 2 Department of Community Program, Dhulikhel Hospital-Kathmandu University Hospital, Kavre, Nepal; 3 Department of Health Law, Policy & Management, Boston University School of Public Health, Boston, MA USA; 4 Department of Obstetrics and Gynecology, Seoul National University College of Medicine, Seoul, Republic of Korea; 5 Department of Obstetrics and Gynecology, Dhulikhel Hospital-Kathmandu University Hospital, Kavre, Nepal; 6 National Assembly Futures Institute, Seoul, Republic of Korea; 7 Department of Thoracic and Cardiovascular Surgery, Seoul National University College of Medicine, Seoul, Republic of Korea

**Keywords:** Obstetric ultrasound, Education, Program evaluation, RE-AIM, LMIC, Global education

## Abstract

**Background:**

Nepal has a high prevalence of congenital anomaly contributing to high infant mortality. Ultrasound, an important tool to detect congenital anomalies and manage maternity-related risk factors, is not properly used in Nepal because Nepali doctors have limited opportunities for learning ultrasound techniques. Hence, we developed and implemented an ultrasound education program from 2016 to 2018. The objective of this study is to evaluate the education program using the Reach, Effectiveness, Adoption, Implementation, and Maintenance (RE-AIM) framework.

**Methods:**

We conducted a mixed-method study to evaluate each component of RE-AIM. The team collected quantitative data from administrative records, tests, surveys, and an online follow-up survey. Qualitative data were collected from individual in-depth interviews at least a year after the program. The proportions, means, and t-tests were used for quantitative data, and thematic coding for qualitative data.

**Results:**

A total of 228 healthcare workers representing 27.3% of the districts of Nepal were reached from 2016 to 2018. The program improved participants’ knowledge (29.3, 8.7, and 23.8 increases out of 100, each year, *p*< 0.001, *n*=85) and self-confidence (0.6, 0.3, 1.3 increases out of 4.0, *p*< 0.01, *n*=111). The participants were highly satisfied with the program (4.2, 4.1, and 4.0 out of 5.0, *n*=162). Among the respondents of the online follow-up survey (*n*=28), 60.7% had used ultrasound in their daily practice after the education program, and a medical institution established an ultrasound training center. The absence of clear accreditation and practical guidelines in ultrasound use were presented as barriers for adoption and maintenance.

**Conclusion:**

The program was successful in improving participant’s knowledge and self-confidence in ultrasound techniques and showed great potential for the adoption and maintenance of the techniques in their practice. Continuous implementation of the program and institutional policy changes to facilitate ultrasound use may increase the ultrasound use and improve ultrasound service quality in Nepal.

## Background

Congenital anomaly contributes to 5–7% of perinatal and neonatal mortality in low- and middle-income countries (LMICs) [1]. The prevalence of congenital anomaly in Nepal is also high (52 per 10,000 children), and 11.1% of neonates die because of congenital anomalies [1–3]. Yet, literature on the care and management of defects in Nepal is limited. Birth defects is an emerging public health problem due to lack of genetic diagnostic methods and inadequacy of other identification methods [4]. Ultrasound is an important noninvasive screening tool that detects congenital anomalies and manages maternity-related risk factors for both morbidity and mortality [5, 6]. The World Health Organization (WHO) recommends pregnant women undergo at least one ultrasound scan before 24 weeks of gestation [7].

Ultrasound has become more widely available for medical professionals in LMICs [8] with decreased equipment costs. However, challenges in improving ultrasound service quantity and quality remain. Workforce shortage is considered a major challenge and a few opportunities for ultrasound training and continuing education exacerbate this challenge. Moreover, several ultrasound training in LMICs does not meet the WHO standards [9].

These challenges are also evident in Nepal. In Nepal, radiologists generally perform ultrasound scans, but Nepal has only 150 radiologists (1 per 185,000 populations), who are largely concentrated around the capital city [10]. This uneven distribution overwhelms ultrasound services in urban areas and thus, service quality control in antenatal care is difficult to achieve [10–12]. Moreover, non-radiologists have limited ultrasound training during their medical school, engaging only a short 15-day rotation in the radiology department. Hence, their ultrasound skill is either basic (limited to acquiring an idea on how machines operate and performing a few procedures with low-quality images) or poor [11, 13].

Previous studies show that intensive ultrasound training courses in LMICs significantly increase knowledge and self-confidence among the radiologists [13, 14]. Hence, the JW LEE Center for Global Medicine at Seoul National University College of Medicine (JW LEE CGM) of the Republic of Korea initiated the intensive ultrasound education program in Nepal since 2016 to promote ultrasound utilization and improve the quality of the practice among both radiologists and non-radiologists through continuous education and experience sharing. In this study, we evaluated the impacts of this education program from 2016 to 2018 using the Reach, Effectiveness, Adoption, Implementation, and Maintenance (RE-AIM) framework [15]. The framework includes an explicit focus on issues, dimensions, and steps in the program design, dissemination, and implementation process, which had been translated and used in different contexts, populations, settings, and cultures [15–18]. We applied the framework to evaluate the effectiveness of knowledge translation, assess broader impacts, and comprehensively understand the limitations, using quantitative and qualitative methods.

## Methods

### Education program development

JW LEE CGM held multiple rounds of needs assessments with key experts from Nepal. Respondents expressed multiple topics of interest such as cutting edge technology in medicine, such as Computed Tomography (CT) and Magnetic Resonance Imaging (MRI); fetal health screening for different systems using ultrasound, CT, and MRI; CT and MRI image and report interpretation; recent developments and issues in ultrasound techniques; detailed demonstration on biometry and other important indicator calculations using different imaging techniques. After exploring the perceived needs, JW LEE CGM invited lecturers/experts, Dhulikhel Hospital-Kathmandu University Hospital (DHKUH), and Nepali partners for educational purposes and created the curriculum based on the needs and availability of lecturers. The course was for two days and titled “The Ultrasound, CT and MRI Education Program for Obstetric, Fetal, Neonatal, and Congenital Anomaly” The course was conducted in collaboration with the Nepali Professional Societies. Table 1 shows the program agenda in 2016, 2017, and 2018. The contents were arranged based on feedback from the previous year’s participants and experts involved (obstetrician-gynecologists [OB-GYNs], pediatricians, radiologists, and hospital leaders). The core component of the program included lectures on obstetric image technology and techniques, case discussions, and experience sharing from both the Korean and Nepali experts. The components of CT and MRI in the program were considered as supplementary sessions because most of the hospitals in Nepal do not perform CT and MRI [19].

**Table 1:** Program agenda of ultrasound, CT, and MRI educational program for obstetric, fetal, neonatal and congenital anomaly in Nepal, 2016, 2017, and 2018

2016	2017	2018
***Day 1 Sessions***		
Basic fetal echocardiography	Fetal CNS	Obstetric USG
Fetal cardiac physiology and transitional circulation	Fetal thorax	Biometry and amniotic fluid volume
Cardiac CT before congenital heart disease surgery	Fetal abdomen	Fetal abdomen
Cardiac MRI for congenital heart disease: when?	Genetic sonography	Fetal genitourinary tract
Fetal CNS	Cervical length and prevention of preterm birth	Fetal echocardiography for congenital heart disease
Doppler USG in obstetrics	Dhulikhel Hospital USG experience	Pediatric echocardiography for congenital heart disease
Experience of anomaly scan in Dhulikhel Hospital	Live-demonstration: fetal screening ultrasound	Dhulikhel Hospital experience in nuchal translucency
Live-demonstration: fetal and neonatal cardiac ultrasound	Live-demonstration: fetal Doppler ultrasound	CT Interpretation of complex congenital heart disease
Live-demonstration: fetal Doppler ultrasound		CT interpretation: extracardiac evaluation in congenital heart disease
Live-demonstration: Fetal screening ultrasound		
***Day 2 Sessions***		
Obstetric USG	Fetal VSD, AVSD	Fetal CNS
Ultrasound of multiple gestation	TOF, pulmonary atresia, TGA	Fetal thorax
Biometry and amniotic fluid volume	CT interpretation of complex congenital heart disease	Fetal Doppler ultrasound
Experience nuchal translucency in Dhulikhel Hospital	Radiologic evaluation: adult congenital heart disease	Genetic sonography

### Participant recruitment for the education program

A national open call for recruitment in the education program was made through various media and professional societies. The main target group of the program was OB-GYNs, pediatricians, and radiologists, but medical doctors from different specialties and non-physician healthcare providers (e.g., nurses, midwives, and paramedical staff) were also recruited. A total of 228 healthcare workers participated in the education program from 2016 to 2018.

### Program evaluation framework

This study utilized the RE-AIM framework, which is a frequently used tool that evaluates multi-organizational and country projects [16]. The RE-AIM framework was applied as a conceptual guide for the study to describe the evaluation process systematically and comprehensively. This framework was selected as it is the most frequently used, and it has been tested with a wide range of interventions. The framework puts special attention on the implementation context [16]. The “Reach” dimension defines the beneficiary population and presents it in numbers covered with demographic and professional characteristics. “Effectiveness” as an impact measurement of an intervention on outcomes, “Adoption” reports settings of intervention agents can be qualitative or quantitative, and “Implementation” describes implementation context and factors influencing implementation. “Maintenance” reports the sustainability of the intervention and factors leading to sustainment. The detailed definitions, indicators, and data sources are presented in Table 2.

**Table 2:** Indicators and data sources for evaluating process and outcomes according to the reach, effectiveness, adoption, implementation, and maintenance framework

Dimensions	Definitions	Indicators	Data Sources (Tools)
**Reach**	An individual-level measure that describes the population the intervention intended to benefit and those that participated or were exposed to the intervention. It is reported as the absolute number, proportion, and representativeness of individuals. (Glasgow(1999, 2018))	Yearly description of participants by demographic characteristics, professional characteristics and geographic coverage.	Enrollment records, initial paper survey
**Effectiveness**	The impact measurement of an intervention on outcomes, including both positive and negative effects. It is presented as an average of the overall effect of outcomes.	Mean difference scores in pre- and post-levels of knowledge	Pre- and post-test
		Mean difference scores in pre- and post-levels of self-confidence	Pre- and post-survey
		Satisfaction scores of healthcare providers who participated the education program	Paper survey
**Adoption (Individual Level)**	Reports settings of a program or policy and intervention agents. This dimension can be reported as a rate or proportion but not limited to those, and qualitative measures can be used.	Percentage of participants reporting the use of the techniques learned	Online survey via email
		Techniques used in current practice	In-depth interview
		What were the participants’ attitudes and beliefs toward the intervention?	
**Implementation**	Describes how the program or policy is delivered, the economics behind a program or policy, and the implementation factors associated with results. The studies’ context implementation dimension focuses on strategies and qualitative approaches used to understand the reasons behind the outcomes.	Description of implementation strategy and processes	Protocol
		Implementing partners	Administrative records
		Inclusion of all components of education program. (Fig. 1)	
**Maintenance (Organizational Level)**	Addresses operational times, the sustainability of the intervention, and factors leading to sustainment. It is reported as a rate or proportion of the recipients that sustained the intervention and institutionalization of practice or policy. Qualitative approaches are also adopted to understand multi-level factors related to sustainment.	Policies and Infrastructure to ensure long-term use of ultrasound	In-depth interview
		Initiatives from partners in Nepal which sustains education program

**Fig. 1:**
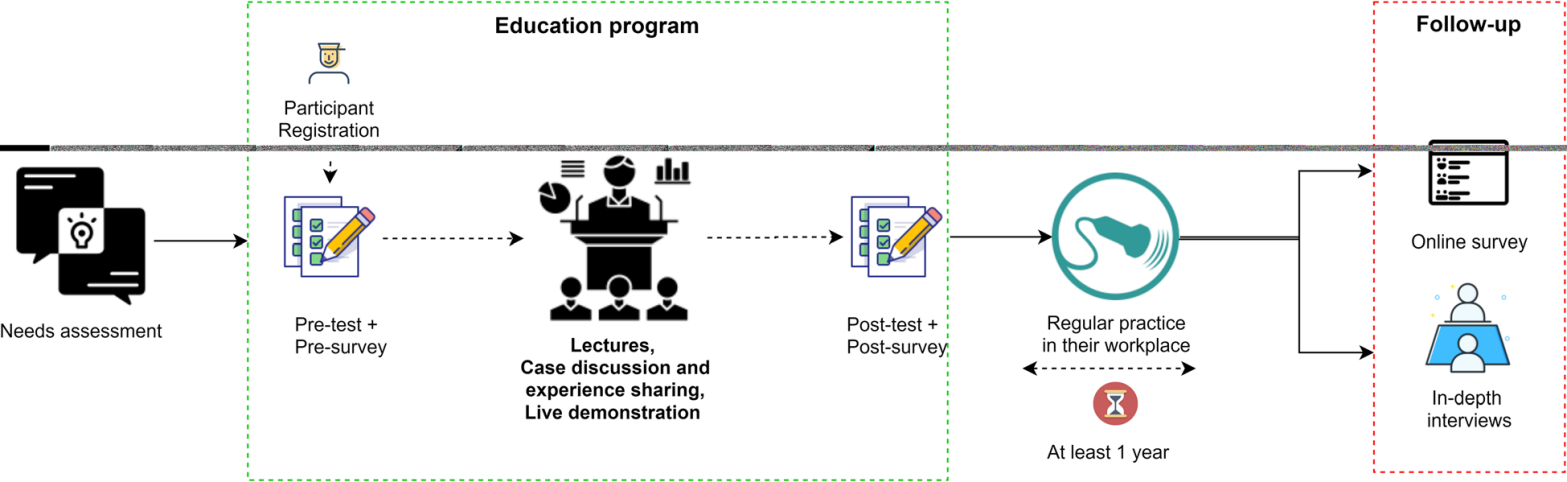
Process of the implementation and evaluation of the education program

### Data collection and outcomes

This study used both quantitative and qualitative methods for evaluation. Administrative data, test data and survey data among participants were collected during the education program (2016–2018), and an online follow-up survey was conducted in 2019. Two members of the research team conducted face-to-face, in-depth interviews in English with the participants and leaders of participating institutions (*n*=11). The interviewees were recruited using convenience sampling.

Outcomes of the study were used in accordance with the components of the RE-AIM framework—Reach, Effectiveness, Adoption, Implementation, and Maintenance. Table 2 shows the details on the process and outcome indicators along with the utilized data sources.

### Analysis

This study used proportions and means for the descriptive analysis and used the mean comparison t-test for difference in pre- and post-test scores for knowledge and pre- and post-survey scores for self-confidence. All quantitative data were analyzed using Stata version 14. For the qualitative data, voice recordings were transcribed and thematically coded using a software R Qualitative Data Analysis (RQDA) package for analysis [20]. PRS coded and formulated the themes based on the inductive method [21], and JK reviewed the codes. Both PRS and JK iteratively refined the subthemes (conceptual labels) and themes (code categories) and linked these with the RE-AIM framework dimensions.

### Ethics

Regarding the tests and surveys, participants verbally consented and voluntarily participated. Written consent was obtained for the online survey and in-depth interviews. Ethical approval was obtained from Institutional Review Boards at the Seoul National University Hospital in the Republic of Korea (SNUH IRB No. 1906–036-1038).

## Results

### Reach

**Fig. 2:**
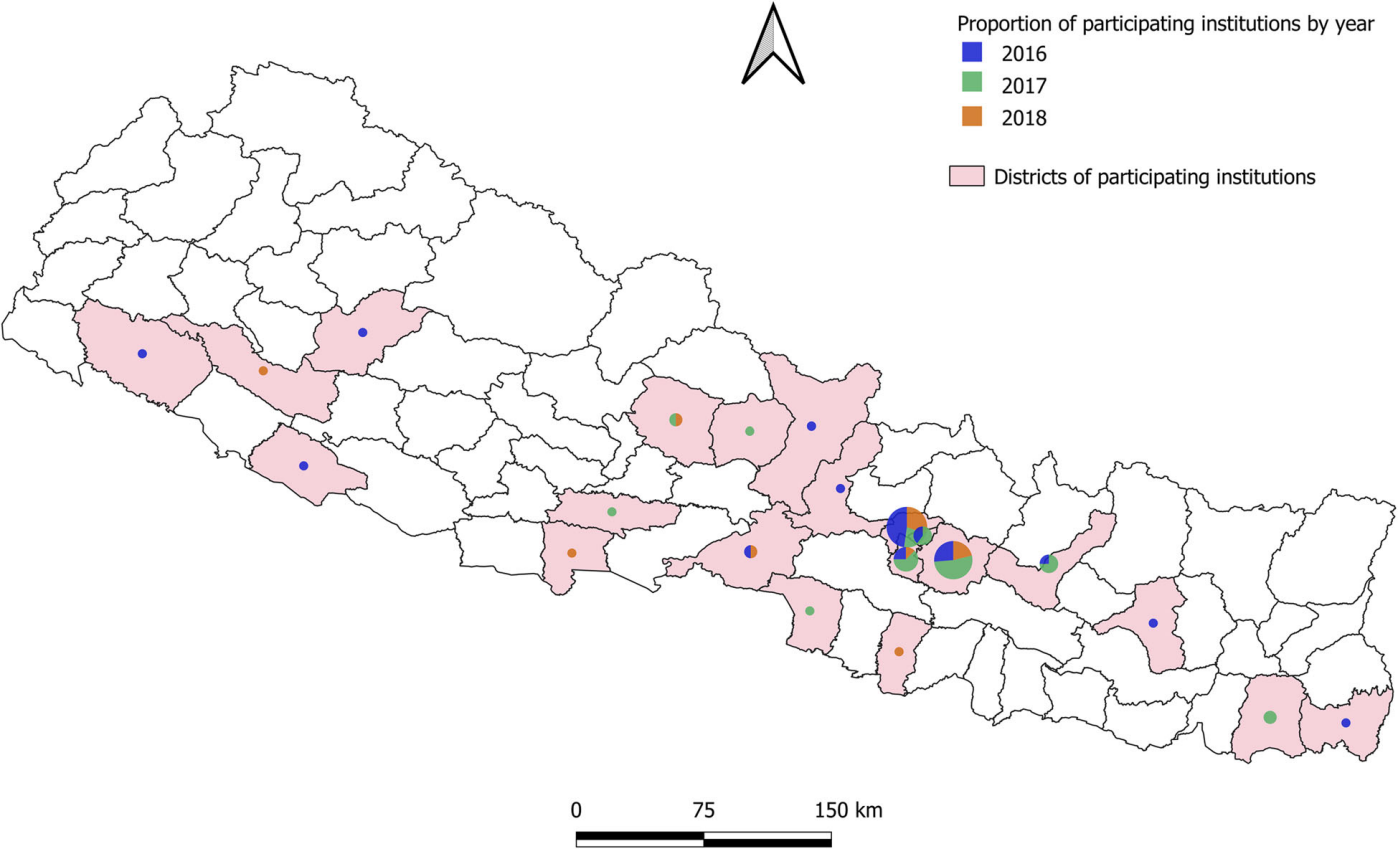
Geographical reach of ultrasound educational program from 2016 to 2018

Table 3 summarizes the demographic characteristics of all participants in the ultrasound education program from 2016 to 2018. Across all years, a total of 228 healthcare workers attended the program, and most of them were aged less than 30 years (46.5, 41.3, and 75.8% in 2016, 2017, and 2018, respectively) and were affiliated with medical colleges (70.7, 54.0, and 81.8% in 2016, 2017, and 2018, respectively), but participants representing other types of institutions such as private hospital, government hospital, healthcare center, and Non-Governmental Organization (NGO) hospital were also present. In case of specialty, all the prioritized groups (OB-GYNs, pediatricians, and radiologists) had similar representation across all 3 years. In terms of geographic coverage calculated by the proportion of districts where the participants’ institution was located (Fig. 2) over the whole districts, one-third of the districts of Nepal (27.3% in 3 years, 16.9% in 2016, 12.9% in 2017, and 10.4% in 2018) were reached. Most of the participating institutes were from capital and its vicinity.

**Table 3:** Characteristics of the participants (2016 to 2018) and online follow-up survey respondents (2019)

Characteristics		2016	2017	2018	Total Participants (2016 to 2018)	Online Follow-Up Survey Respondents (2019)	***P***-Value^****^
Total N (%)		99 (100)	63 (100)	66 (100)	228 (100)	28 (100)	
Sex	Male	65 (65.7)	43 (68.3)	26 (39.4)	134 (58.8)	22 (78.6)	0.068
	Female	34 (34.3)	20 (31.8)	40 (60.6)	94 (41.2)	6 (21.4)	
Age	18–29	46 (46.5)	26 (41.3)	50 (75.8)	122 (53.5)	10 (35.7)	< 0.001
	30–39	37 (37.4)	25 (39.7)	10 (15.2)	72 (31.6)	3 (10.7)	
	40+	14 (14.1)	10 (15.9)	5 (7.6)	29 (12.7)	14 (50)	
	Missing	2 (2.0)	2 (3.2)	1 (1.5)	5 (2.2)	1 (3.6)	
Type of Institution	Medical college	70 (70.7)	34 (54.0)	54 (81.8)	158 (69.3)	14 (50)	0.039
	Others^*^	26 (26.3)	29 (46.0)	9 (13.6)	64 (28.1)	14 (50)	
	Missing	3 (3.0)	0 (0.0)	3 (4.6)	6 (2.6)	0	
Specialty	OB-GYN*****	26 (26.3)	12 (19.1)	12 (18.2)	50 (21.9)	8 (28.6)	
	Pediatrician	16 (16.2)	7 (11.1)	6 (9.1)	29 (12.7)	2 (7.1)	0.762
	Radiologist	19 (19.2)	13 (20.6)	4 (6.1)	36 (15.8)	4 (14.3)	
	Others^**^	38 (38.4)	31 (49.2)	41 (62.1)	110 (48.2)	14 (50)	
	Missing	0 (0.0)	0 (0.0)	3 (4.6)	3 (1.3)	0	
Experience	< 3 years	29 (29.3)	44 (69.8)	38 (57.6)	111 (48.7)	14 (50.0)	0.179
	≥3 years	24 (24.2)	18 (28.6)	17 (25.8)	59 (25.9)	14 (50.0)	
	Missing	46 (46.5)	1 (1.6)	11 (16.7)	58 (25.4)	0	
Position	Junior	59 (59.6)	31 (49.2)	45 (68.2)	135 (59.2)	50 (50.0)	< 0.001
	Senior	29 (29.3)	17 (27.0)	7 (10.6)	53 (23.2)	50 (50.0)	
	Others^***^	9 (9.1)	14 (22.2)	13 (19.7)	36 (15.8)	0	
	Missing	2 (2.0)	1 (1.6)	1 (1.5)	4 (1.8)	0	

***Note***: ^*^Private hospital and clinics, non-governmental organization hospital, government health institutions^**^Emergency, nursing, surgery

^***^Director

^****^P-value for chi-squared test of independence between the total participants and online survey population

^*****^OB-GYN: obstetrician-gynecologist

### Effectiveness

Table 4 indicates the changes in knowledge and self-confidence before and after the education and overall satisfaction of the program. The mean post-test scores were 78.2, 50.1 and 59.3 out of 100 from 2016 to 2018. The test scores were significantly increased by 29.3, 8.7, and 23.8 (*p*< 0.001) compared to pre-test scores. Similarly, the mean post-self-confidence scores were 3.0 in all three years which were increased significantly by 0.6, 0.3, and 1.3 out of 4 points (*p*< 0.01) compared to pre-self-confidence scores. The participant’s overall satisfaction score of the education program was high (4.2, 4.1, and 4.0 out of 5 in 2016, 2017, and 2018, respectively) across all 3 years.

**Table 4:** Effects of ultrasound education program for obstetric, fetal, neonatal and congenital anomaly in Nepal, 2016, 2017, and 2018 on self-confidence, knowledge, and satisfaction

	2016					2017					2018				
	N	Pre	Post	Diff	P	N	Pre	Post	Diff	P	N	Pre	Post	Diff	P
Test Score	27	48.4 (10.6)	78.2 (13.0)	29.3 (15.0)	< 0.001	26	41.4 (12.4)	50.1 (12.9)	8.7 (8.9)	< 0.001	32	35.5 (8.5)	59.3 (11.1)	23.8 (9.1)	< 0.001
Self-Confidence	39	2.4 (0.8)	3.0 (0.4)	0.6 (0.7)	< 0.001	32	2.7 (0.5)	3.0 (0.5)	0.3 (0.5)	0.002	40	1.7 (0.5)	3.0 (0.8)	1.3 (0.9)	< 0.001
Satisfaction	56		4.2 (0.5)			55		4.1 (0.4)			51		4.0 (0.4)		

### Adoption

Approximately 12% (*n*=28) of the program participants responded to the online follow-up survey. Approximately 50% (*n*=14) of the online follow-up survey respondents participated for the first time in 2016, 36% (*n*=10) in 2017, and the remaining 14% (*n*=4) in 2018. Among the online follow-up survey respondents, 60.7% (*n*=17) indicated that they were currently using ultrasound in their daily practice (Table 5). When we divided the respondents by specialty, we found that most of the radiologists (75%, *n*=3) and other specialties (71.4%, n=10) and half of OB-GYNs (50%, n=4) began to use ultrasound in their daily practice after participating in the education program. None of the pediatricians who participated in the survey have reported the use of ultrasound.

**Table 5:** Online follow-up survey results regarding ultrasound usage

Using Ultrasound in Current Practice	N=28 (%)
Overall (all subgroups)	17 (60.71)
Radiologist	3 (75.00)
OB-GYN	4 (50.00)
Pediatrician	0 (0.00)
Others	10 (71.42)
**Changed Practice after Training Program**	
Yes	14 (82.35)
No	3 (17.65)
**Techniques Currently Used**	
Fetal CNS	10 (58.82)
Fetal Doppler	8 (47.05)
Fetal echo	3 (17.65)
Fetal thorax	1 (5.89)
Biometry	2 (11.76)
Cervical length	2 (11.76)
Fetal abdomen	2 (11.76)
Genetic sonography	0 (0)
Pediatric echo	0 (0)
Fetal genitourinary	0 (0)

^*^OB-GYN: obstetrician-gynecologist

Table 6 illustrates the in-depth interview results including major limitations of ultrasound utilization. The lack of availability of ultrasound machines and lower competency levels due to lack of regular training and practice were identified as the major challenges for the adoption of ultrasound. Moreover, restrictive institutional and national policies were also identified as limitations. Radiologists performed ultrasound in their daily practice, whereas non-radiologists used ultrasound during emergencies and for confirmation of their own diagnosis. Some non-radiologists from institutions that promote the use of ultrasound reported performing a high volume of ultrasound in their practice after participating in the education program. Fetal neurosonogram and fetal Doppler techniques were widely used (58.8 and 47.1% of ultrasound users, respectively), whereas genetic sonography, pediatric echo, and fetal genitourinary techniques have not been used. A non-radiologist expressed concerns on overuse of ultrasound, “There is a high dependency on ultrasound, leading to ultrasound overuse, in conditions where other diagnostics methods were sufficient.” A radiologist also had a concern regarding ultrasound misuse as “ultrasound can be used for commercial purposes.”

**Table 6:** Themes, subthemes, and quotations for adoption and maintenance dimensions

Themes and Definitions	Subthemes and Definitions	Quotes
**Adoption:** Use of the ultrasound in daily practice by the participants		
**Ultrasound use:** Participants expressed they were using ultrasound at least at some level in all targeted specialties (OB-GYN, pediatrician, and radiologist), independently or with support from the radiology department	**Process/events:** General practice of ultrasound and occasions of use in the participant’s institution	“...In our institution, routine ultrasound is performed by the radiologist. However, for screening purposes such as emergency ultrasound, it has been performed by other healthcare workers in other departments including emergency department and obstetrics and gynecology department” – Pediatrician
		“To determine early pregnancy, the general condition of the fetus during late pregnancy, the appropriate location of the placenta, the alignment of the uterus, ultrasound in gynecology is required. Moreover, to confirm the presence of tumors and fibrocystic lesions or latencies, performing ultrasound is an essential part of our practice.” – OB-GYN “Cranial ultrasound, regular echocardiography, and abdominal ultrasound including renal scan are performed regularly to screen and to diagnose more complicated cases and to immediately respond to emergency department and intensive care unit care” – Pediatrician
	**Frequency:** Number of cases participants directly perform ultrasound	“….Hence possibly in a day we examine approximately 55 patients; among them, I examine possibly 10 patients daily…” – OB-GYN “I perform ultrasound in nearly 50% of admitted cases and 10 to 20% of outpatient department cases” –Pediatrician “In the past, ultrasonography and sonography were considered as the standard modalities, but they were mostly unavailable. However, today, these modalities are regularly performed in clinical practice in wards, outpatient departments, and even in the emergency department. It has been well accepted by the patients as well.” – OB-GYN
**Perspective toward educational program:** Respondents expressed their views and observations regarding the educational program and changes attributed to the program	**Use of ultrasound in regular practice:** Use of ultrasound in regular practice attributed to the program	“...nowadays, everyone is competent to perform ultrasound regularly. Moreover, healthcare workers already decide on the dose of inotropes based on echo results.” – Pediatrician “...the training has addressed the topics we dealt in daily practice, improving confidence in establishing clinical decisions…” – OB-GYN
	**Correction in practice:** Previously used techniques were corrected or improved based on the program	“The ultrasound was already used during the past years, but specific guidelines in performing ultrasound to assess congenital anomalies were not yet established... after attending the course, we already properly performed ultrasonography. The knowledge obtained was shared with the resident students as well, and they significantly improved.” – OB-GYN
	**Negative perspectives:** The negative views toward the program and its potential negative outcomes	“...This type of program has several controversies, specifically in Nepal...there is no system of accreditation. It encourages the use of ultrasound without any proper certification. There is potential misuse of ultrasound. There is no proper follow-up.” – Radiologist
**Challenges in the use of ultrasound:** The participants expressed reasons for not using ultrasound when they first start or for the optimal use	**Lack of ultrasound machine:** Participants mentioned that there are no ultrasound machines or fewer ultrasound machines	“The gynecologists are not performing ultrasonography in their clinics because of the unavailability of any ultrasound machine, including my clinic. In private hospital or clinic setup, it is expensive to keep an ultrasound machine.” – OB-GYN “Initially, lack of ultrasound equipment was one of the challenges; however, currently, it is now gradually addressed; for instance, the Seoul National University donated a decent echo machine, and we are significantly grateful for that. We are using this machine for pediatric echocardiography and transcranial scans. Gradually, our department is also equipped with few other machines.” –Pediatrician “Every single day, one doctor has to perform 10 to 15 congenital anomaly scans; hence, it is a difficult job. If the cases were significantly few, that is, 4 to 5 per day, it would have been covered by the current infrastructure.” –Radiologist
	**Lower competency:** Participants expressed low competency and nonconfidence in performing ultrasound on their own	“During emergency conditions in high-risk pregnancy, OB-GYNshould be able to perform ultrasonography, but considering the insufficient training programs for OB-GYN, performing ultrasonography is the major cause of OB-GYN’s lower competencies.” – OB-GYN
**Perspective on ultrasound use:** How individual observed the use of ultrasound in the current context	**Overuse of ultrasound:** Use of ultrasound where it was not absolutely required. High dependency on the ultrasound giving lower importance to other aspects	“We over diagnosed several diseases because of ultrasound. However, sometimes, we try to overlook our clinical practice and our clinical skills similar to what we do when using the stethoscope and when performing palpitations, and our clinical practice is sometimes insufficient, and we depend on the ultrasound machines. Hence, I think that sometimes we have to assess the risks and benefits. It has changed our decision-making skills.” – Pediatrician
	**Misuse of ultrasound:** Use of ultrasound guided other intentions	“The use of ultrasound for inappropriate cases and for commercial purposes. It is easy to obtain 1000 rupee if you use ultrasound. People undergo 15 days of training and can subsequently open their ultrasound service center. This is considered a significant unethical issue.” - Radiologist
	**Important diagnostic tools:** Participants express ultrasound as the important tool for diagnosis and investigation	“Ultrasound is one of the most important diagnostic or investigative tools.”
	**Improved quality of patient management:** Participants associate ultrasound use with improved patient management	“When performing clinical practice and when using the ultrasound machines to confirm our clinical diagnosis, disease diagnosis and management is significantly easier and accurate. Hence, I think ultrasound machine has changed our decision-making skills and improve our patient care.” - Pediatrician
**Maintenance:** Institution have policies (also includes national), infrastructures, and initiatives to sustain the use of ultrasound		
**Infrastructure maintenance:** The participants expressed recent developments or improvements in instruments, spaces, and human resource empowerment	**Availability of ultrasound machines:** Participants mentioned about the upgrade or addition of ultrasound machines	“In Nepal, we have a relatively good number of ultrasound machines that are available not only in hospital but also in outreach centers such as in remote areas. For the past 2 years, I think we added 20 ultrasound machines.” –Radiologist “Our hospital has provided ultrasound machines in our wards, labor rooms, emergency rooms, and in all areas where we might need an ultrasound machine.” – OB-GYN
	**Competency of human resources:** Participants mentioned the availability of trained human resources or doctors who can perform ultrasound and opportunity for getting human resources trained	“….the consultants and junior doctors in my department are competent in performing basic ultrasonography by themselves.” – OB-GYN “Sometimes, when we encountered difficult cases, we used to discuss with healthcare professors who were able to undergo trainings from Korea via emails and discuss the ultrasound images.” – Pediatrician
**Policy:** The participants expressed national or institutional policy including formal written policy or informal practice-related policy	**Institutional policy to encourage practice:** Participants mentioned about written policy or unwritten but understandable policy that encourages the use of ultrasound	“There is no written protocol regarding the use of ultrasound, but limitations regarding its use are not yet established. When we train the resident doctors, they perform the ultrasound scan for inpatient children during the evening whenever they are free.” – Pediatrician “There is no written guideline stating that ultrasonography should be performed by a gynecologist, but in the department, the use of ultrasound is encouraged. During the department meeting, we encourage colleagues to perform ultrasonography to establish the diagnosis of a disease.” – OB-GYN “When I joined the residence here, a consultant doctor (teacher) said that I will be doing transvaginal sonography gradually and will be diagnosing the intrauterine pregnancy.” – OB-GYN
	**Restriction in practice by policy:** Participants mentioned about restriction in use of ultrasound in practice or incorporation in formal guidelines by policy	“We do not have any written (formal) document indicating ultrasound scans need to be performed by obstetricians, but as part of our examinations, we perform it.” – OB-GYN “The eligibility of a healthcare worker to perform ultrasound and the guidelines used to certify a healthcare worker regarding the use of ultrasound are not yet established in Nepal. Currently, any healthcare worker (including staff nurse, health assistant, and even Ayurvedic doctor) with an ultrasound machine can perform ultrasonography. There is no proper system of accreditation.” – Radiologist “My findings will not be as strong as it is when evaluated by a radiologist for documentation, even if we can identify the problem, we need to refer our findings to a radiologist.” – OB-GYN
**Local initiatives:** The participants expressed about how they are taking forward the ultrasound knowledge dissemination and promotion		“An ultrasound training center had been established in Dhulikhel Hospital with good infrastructures. Separate department had been established to handle this. The institution has started the ultrasound training on their own and also has expanded their collaborations. The curriculums and modules for various levels of ultrasound education had been developed, and education is being provided continuously.” – Radiologist

^*^OB-GYN: obstetrician-gynecologist

All the interviewees expressed positive attitudes toward the education program. They attributed the program participation to the use of ultrasound in their workplaces. They also expressed that the program helped them improve their use of ultrasound in their practice. For future direction of the program, the interviewees suggested that certification and accreditation policy, educational session on the misuse of ultrasound, and online follow-up after the program should be implemented. A radiologist expressed, “...there is no system of accreditation. It encourages using ultrasound without any proper certification. There is potential misuse of ultrasound. There is no proper follow up.”

### Implementation

The program was developed and implemented in collaboration with the DHKUH and Nepal Society of Obstetricians and Gynaecologists. The Ultrasound Society of Nepal joined as a co-organizer, whereas the Nepal Radiologists’ Association and Nepal Paediatric Society endorsed and promoted the education program. The Nepal Medical Association accredited the continuing medical education credits based on curriculum and content. The involvement of Nepal medical societies and associations ensured a wide acceptance of the program.

Figure 1 shows the process of implementation. The program had four major components: lectures, case discussion and experience sharing, live demonstration, and program evaluation (Fig. 1). The major difference in the program implementation across the years was the exclusion of live demonstration sessions in 2018 based on the feedback and logistic difficulties. Furthermore, major differences in the program implementation modality were not observed.

### Maintenance

The Interviewees mentioned that ultrasound use is still lagging due to unclear policy and high cost for the ultrasound machines for private hospitals (Table 6). Few of the institutions have developed infrastructure such as purchasing ultrasound machines and trained human resources and fostered networks for the discussion of cases via tele chats. Both radiologists and non-radiologists expressed dissatisfaction with the current policy in terms of ambiguity in accreditation and the absences of practical guidelines for ultrasound use by non-radiologist. A radiologist expressed, “The eligibility of a healthcare worker to perform ultrasound and the guidelines used to certify a healthcare worker regarding the use of ultrasound are not yet established in Nepal. Currently, any healthcare worker (including staff nurse, health assistant, and even Ayurvedic doctor) with an ultrasound machine can perform ultrasonography. There is no proper system of accreditation.” Similarly, a non-radiologist practicing ultrasound expressed, “My findings will not be as strong as it is when evaluated by a radiologist for documentation. Even if we can identify the problem, we need to refer our findings to a radiologist.” These types of ambiguity and inefficiency in national policy on accreditation was expressed as the major limitation of ultrasound utilization by non-radiologists. The high cost of an ultrasound device makes it difficult for private hospitals to use ultrasound prohibiting them to practice and maintain the skills that they have learned. Only one institution established an ultrasound-training center and initiated regular training for doctors and healthcare workers, partly stimulated by the education program.

## Discussion

To improve the ultrasound utilization and the service quality of maternal and child health in Nepal, the JW LEE CGM and the DHKUH have conducted “The Ultrasound, CT and MRI Education Program for Obstetric, Fetal, Neonatal, and Congenital Anomaly” in collaboration with the Nepali Professional Societies. This study evaluated the 2-day intensive ultrasound education program conducted in Nepal from 2016 to 2018 in terms of its Reach, Effectiveness, Adoption, Implementation, and Maintenance using the RE-AIM framework. This study showed that the ultrasound education program was effective in improving the knowledge and self-confidence of physician and non-physician participants. However, only half of the online survey respondents indicated that they used learned ultrasound techniques in their daily practices after participating in the education program. This study also observed significant heterogeneity in ultrasound utilization by site and across all healthcare professions in Nepal. Changing regulations at institutional and national levels to facilitate use of ultrasound, and improve availability of ultrasound devices may increase the adoption and maintenance of the ultrasound in practice.

The ‘Reach’ dimensions of the program were satisfactory in terms of participants’ diversity in specialty and affiliation. Geographically, however, the program reached approximately 27% of the districts in Nepal. This may be due to the uneven distribution of ultrasound services across healthcare facilities and providers in Nepal. Unavailability of machines and human resources might have resulted in lower geographical coverage. A previous study on the availability of technology for trauma care in Nepal, which accessed 56 small hospitals (with a bed capacity of 30 to 100) and 29 large hospitals, reported that ultrasound was not available in any of accessed small hospitals [19]. The health facility survey in 2015 showed that only 21.1% of the district-level hospitals had basic equipment and approximately half of the sanctioned position of medical officers and Doctor of Medicine in General Practice (MD-GP) were completed [12].

This study shows that the program was effective in increasing the healthcare workers’ knowledge and self-confidence in using ultrasound. The participants were also satisfied with this program. We observed a slight decrease in the satisfaction score from 2016 to 2018, but the mean scores were above 4 out of 5. The education program on ultrasound with several implementation modalities and durations with the use of pre- and post-test to assess knowledge also resulted in significant improvement in knowledge immediately after the programs [9, 13, 22, 23]. A previous study from Nepal on point-of-care ultrasound training also reported improvement in post-test scores in the 1-day training, which was mostly participated by doctors without prior experience [13]. Further, Shaffer et al. (2017) reported that the self-reported confidence for four specific skills increased among the participants at the end of the short-term (5-day) training program on bedside emergency ultrasound [24]. We removed the live demonstration component in 2018 due to technical and logistical difficulties, but it did not lead to a significant decrease in the three components of the ‘Effectiveness’ dimension.

This study found a mid-level (60%) of adoption at the individual level via online follow-up survey. The non-adoptees emphasized that further policy changes to encourage the use of ultrasound by non-radiologists along with infrastructure development are considered the major limitations in the use of ultrasound. Setting up and maintenance of the machines can also be significantly challenging in rural areas of Nepal [11]. Another interviewee emphasized the uncertainty in the current policies, implying the prohibitive use of ultrasound by the non-radiologists. The clinical guidelines do not also explicitly mention the protocols regarding the use of ultrasound by doctors in the non-radiology departments. A clear scope on the use of ultrasound and eligibility of performance may facilitate ultrasound utilization and service quality.

The institutional-level ‘Maintenance’ dimension of the RE-AIM framework had mixed results. Despite the challenges and limitations regarding the changes in policy and infrastructure development, one of the participating institutions in Nepal had established an ultrasound training school, and they collaborated with different specialties to develop courses for training physician and non-physician healthcare providers. However, some organizations are still having trouble in formulating clear institutional policies on ultrasound use by non-radiology doctors. The establishment of the ultrasound training center can be considered a crucial step in the sustainability of interdisciplinary educational programs.

This study has several limitations. First, we used convenience sampling methods for in-depth interviews because of time constraints and insufficient resources to travel beyond Kathmandu and its vicinity. The second limitation was low participation rates in evaluations. Participation in the evaluation was voluntary, and many of the registered participants were allowed to select sessions and skip tests and surveys. We offered flexibility since the second day of the training took place during the workday. Many participants were not able to arrange a leave for the day. In the case of the online survey, a low response rate was expected, thus we tried to mitigate this by sending three reminder emails. Due to low participation rates, the findings may have induced selection bias. Finally, the evaluation of self-confidence and use of ultrasound was based on self-reporting, which may cause social desirability bias. Despite these limitations, this study provides evidence that the education program can be used as a platform for sharing knowledge and experiences across healthcare providers with diverse backgrounds.

## Conclusion

Our study found that the ultrasound education program effectively improved the knowledge and self-confidence of participants in practicing ultrasound. The program also had a positive impact on ultrasound utilization by encouraging participants to use ultrasound; however, determining the influencing institutional environment that facilitates ultrasound utilization and improves service quality was still considered challenging due to the current regulations. Future studies are required to comprehensively understand the various elements influencing the use of learned knowledge and acquired skills that translates into actual practice that significantly affects the health at the population level.

## Data Availability

The datasets generated and/or analyzed during this study are available from the corresponding author upon reasonable request.

## Abbreviations

LMICs: Low- and Middle-income Countries
WHO: World Health Organization
JW LEE CGM: JW LEE Center for Global Medicine at Seoul National University College of Medicine
RE-AIM: Reach, Effectiveness, Adoption, Implementation, and Maintenance
DHKUH: Dhulikhel Hospital Kathmandu University Hospital
OB-GYN: Obstetrician-gynecologist
CT: Computed Tomography
MRI: Magnetic Resonance Imaging
RQDA: R Qualitative Data Analysis
NGO: Non-Governmental Organization
SNUH: Seoul National University Hospital in the Republic of Korea
MD-GP: Doctor of Medicine in General Practice
